# Strain energy analysis of floor heave in longwall gateroads

**DOI:** 10.1098/rsos.180691

**Published:** 2018-08-08

**Authors:** Meng Wang, Dongjie Zheng, Kewei Wang, Wenfeng Li

**Affiliations:** 1 School of Energy Science and Engineering, Henan Polytechnic University, Jiaozuo, Henan, People's Republic of China; 2 State Key Laboratory of Coal Resources and Safe Mining, China University of Mining and Technology, Xuzhou, Jiangsu, People's Republic of China; 3 Department of Energy and Power Engineering, Xuhai College, China University of Mining and Technology, Xuzhou, Jiangsu, People's Republic of China

**Keywords:** longwall mining, strain energy, floor heave, post-failure rocks, gateroads

## Abstract

Floor heave in longwall gateroads is a severe issue that affects mining safety and efficiency. Researchers, however, have limited understanding on the floor heave mechanism because the deformation of post-failure rocks in the floor was seldom considered previously. In this study, we developed a theoretical model using the strain energy theory to investigate the post-failure deformation of rocks. This model was validated before being implemented into a numerical modelling package, FLAC^3D^, for floor heave analysis. Based on a case study of a longwall entry employing a stiff–yield pillar configuration, we observe that massive floor heave occurs at the entry rib that takes less loads (yield pillar) and eventually propagates towards the other rib bearing a significant amount of loads (stiff pillar). This observation sheds light on the floor heave mechanism in longwall gateroads and has major implications for coal mine ground control.

## 1. Introduction

Coal is the major energy source in China. It is reported that, in 2012, coal consumption has reached 1873.3 million tonnes of oil equivalent, which accounts for 68% of the total energy consumption in China [1]. To extract the coal seams from the subsurface, longwall mining is widely used because of its efficiency. However, longwall mining involves a wide range of issues, such as rock bursts and coal bumps [2–6], pillar instability [7–13], significant surface subsidence [14] and other environmental issues [15]. Among those issues, floor heave in longwall gateroads has drawn increasing attention due to its significant effect on mining safety and efficiency. In-mine measurement indicates that the magnitude of floor heave in the gateroads may reach up to 900–1200 mm, depending on the geological and mining conditions [2,16]. Severe floor heave significantly reduces the accessibility of the entries and, in some cases, causes longwall panels to be abandoned.

Researchers have studied the mechanism of floor heave in longwall gateroads. Some researchers stated that the floor heave occurs when the loads transferred by the pillars exceed the bearing capacity of the pillar foundation (immediate floors) [16]. Researchers further divided the floor under a pillar foundation into three distinct zones: active zone, radial shear zone and passive zone [17]. The deformation in the active zone and the radial shear zone gradually causes the massive failure in the passive zone, resulting in the severe floor heave in entries [18,19]. Researchers have also developed many formulae, considering the influence of moisture content and long-term loading on floor rocks, to characterize the bearing capacity of the immediate floor under specified geological and mining conditions [20–26]. These studies significantly contribute to our understanding on the floor heave mechanism in longwall gateroads.

Different mechanisms of the floor heave in longwall gateroads have also been proposed. Based on mechanical analysis, Kang & Lu [27] stated that bending of the immediate floor layer stands to be the vital reason for the floor heave in gateroads. From the investigation of various cases with different geological and mining conditions, Jiang & Lu [28] classified the mechanism into four basic categories: squeezing, bending, swelling and shearing. Moreover, Wang [29] conducted a comprehensive study on the mechanism of floor heave in entries employing the stiff, yield and artificial pillars; he reported that the immediate and the main floor layers showed different tendencies towards heave. These studies have also greatly improved our understanding of the floor heave mechanism.

However, the post-failure deformation of the floor rocks, during the longwall retreating operations, has not been properly analysed for the characterization of floor heave. In most cases, the immediate floors of coal seams are weak rocks, such as mudstone, shale and claystone. These rocks are likely to fail at an early time of mining operations, which suggests that the floor heave in longwall gateroads is mainly caused by the deformation of the post-failure rocks in the immediate floor. Unfortunately, this was not well discussed previously due to the lack of a proper indicator for characterizing the post-failure behaviour of rocks.

From the rock strain-energy point of view, floor heave results from the work done by the mining-induced loads that are exerted on the floor rocks. The work done is consumed by the stored elastic energy and the failure energy of the floor rocks [30–36]. The stored elastic energy represents the recoverable energy upon removal of the loads, while the failure energy includes the dissipated energy in the form of plastic deformation and the released energy during cracking. Therefore, we may use these strain energy terms to characterize the deformation of the post-failure rocks for a better understanding of the floor heave mechanism.

In this paper, we correlate the strain energy change with the deformation of the post-failure rocks to evaluate the mechanism of floor heave in longwall gateroads. For this purpose, we first derive an analytical model to calculate the stored elastic energy and the failure energy during rock deformation, based on which we propose an indicator to investigate the post-failure behaviour of rocks. We validate the analytical model using laboratory measurements. The developed strain energy model is finally implemented in a numerical simulation package, FLAC^3D^, to investigate the floor heave mechanism in longwall gateroads.

## 2. Strain energy model

### 2.1. Background of strain energy

**Figure 1.**
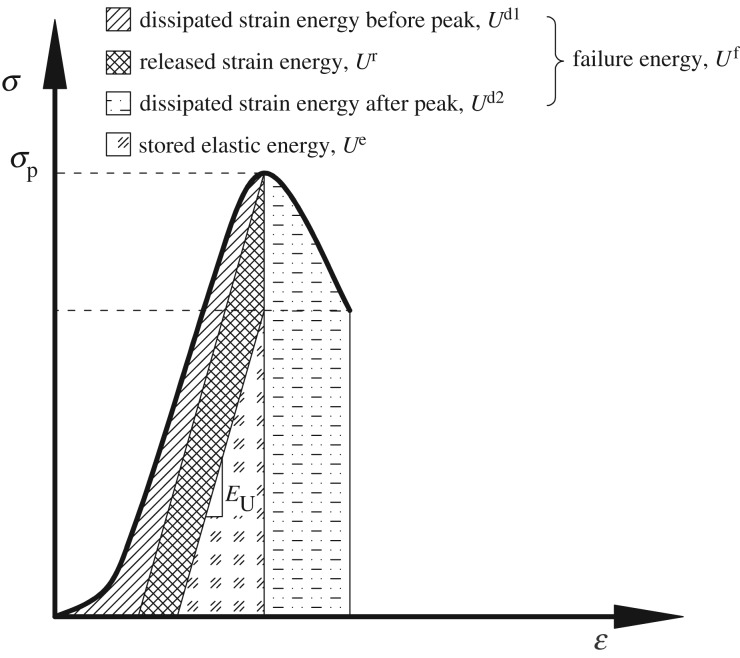
Energy terms per unit volume of rocks under loading condition. Total work done, *W*, is consumed by the stored elastic energy (*U*^e^) and the failure energy (*U*^f^). Note that *E*_U_ represents the unloading modulus of the rock.

As stated preciously, the deformation of rocks is basically caused by the work done by external loads. If we ignore the thermal energy that is created by the sliding on the generated cracks during rock deformation, the work done by the external loads can be classified into stored elastic energy and failure energy according to the following equation [33,35,37]:

2.1 $$W=U^{e}+U^{f},$$

where *W, U*^e^ and *U*^f^ are the total work done, stored elastic energy and failure energy, respectively, during the rock deformation. The relation among these quantities per unit volume of rocks is shown in figure 1. The failure energy (*U*^f^) includes the dissipated energy before the peak (*U^d^*^1^), the dissipated energy after the peak (*U^d^*^2^) and the released elastic energy (*U*^r^).

In terms of principal stresses and strains, *W*, *U*^e^, and *U*^f^ per unit volume of rocks can be calculated by [33,38]

2.2 $$\begin{array}{rl} W & =\sum \int_{0}^{\varepsilon_{i}}\sigma_{i}\text{d}\varepsilon_{i}, \end{array}$$

2.3 $$\begin{array}{rl} U^{e} & =\sum \frac{1}{2}\sigma_{i}\varepsilon_{i}^{e}\text{, } \end{array}$$

2.4 $$\begin{array}{rl} \varepsilon_{i}^{e} & =\frac{1}{E_{i}^{u}}[\sigma_{i}-\nu_{i}(\sigma_{j}+\sigma_{k})] \end{array}$$

2.5 $$\begin{array}{r} \text{and}\;\;U^{f}=W-U^{e}, \end{array}$$

where $\sigma_{i},\varepsilon_{i}^{e},E_{i}^{u},\text{and }\nu_{i}$ (*i*  =  1, 2, 3) are the principal stresses, recoverable elastic strains, unloading moduli and Poisson's ratio, respectively.

### 2.2. Strain energy calculation

**Figure 2.**
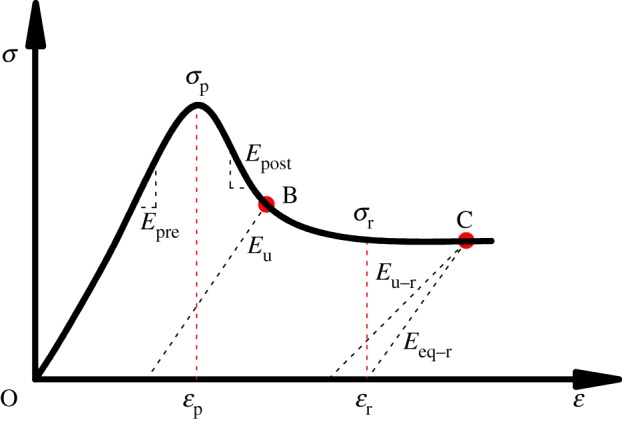
Complete stress–strain curve of rocks for the strain energy calculation.

The stress–strain behaviour of rocks is simplified to facilitate the calculations of strain energy in unit volume of rocks. The deformation of rocks is divided into three distinct regions, as shown in figure 2.

#### 2.2.1. Linear elastic region (*oε*_p_)

The linear elastic region demonstrates the pre-peak portion of the rock deformation. In this region, the work done is totally transformed into the stored elastic energy. Thus, we have *W*  =  *U*^e^ and *U*^f^  =  0. Note that *W* and *U*^e^ can be calculated using equations (2.2) to (2.4). Additionally, the fraction of the failure energy *U*^f^ in the total work done is

2.6 $$f^{f}=\frac{U^{f}}{W}=\frac{U^{f}}{U^{e}+U^{f}}=0.$$

#### 2.2.2. Post-failure region (*ε*_p_*ε*_r_)

This region mainly represents the softening portion in the stress–strain curve of the rock deformation. In this region, the work done is consumed by both the stored elastic energy and the failure energy. Suppose point B is in this region (figure 2); then the following equations exist:

2.7 $$\begin{array}{rl} U^{\mathrm{pre}} & =\frac{1}{2E_{\mathrm{pre}}}[\sigma_{1p}^{2}+\sigma_{2p}^{2}+\sigma_{3p}^{2}-2\nu (\sigma_{1p}\sigma_{2p}+\sigma_{1p}\sigma_{3p}+\sigma_{2p}\sigma_{3p})], \end{array}$$

2.8 $$\begin{array}{rl} U^{\mathrm{post}} & =\frac{1}{2E_{\mathrm{post}}}\left[\begin{array}{c} \sigma_{1p}^{2}+\sigma_{2p}^{2}+\sigma_{3p}^{2}-2\nu (\sigma_{1p}\sigma_{2p}+\sigma_{1p}\sigma_{3p}+\sigma_{2p}\sigma_{3p})\\ -(\sigma_{1B}^{2}+\sigma_{2B}^{2}+\sigma_{3B}^{2})+2\nu (\sigma_{1B}\sigma_{2B}+\sigma_{1B}\sigma_{3B}+\sigma_{2B}\sigma_{3B}) \end{array}\right] \end{array}$$

2.9 $$\begin{array}{rl} \text{and}\;\;\;U^{e} & =\frac{1}{2E_{U}}[\sigma_{1B}^{2}+\sigma_{2B}^{2}+\sigma_{3B}^{2}-2\nu (\sigma_{1B}\sigma_{2B}+\sigma_{1B}\sigma_{3B}+\sigma_{2B}\sigma_{3B})], \end{array}$$

where *U*^pre^ and *U*^post^ are the strain energies in the pre-peak and post-peak regions, respectively; *U*^e^ is the stored elastic energy; *E*_pre_ and *E*_post_ are the loading and post-peak moduli, respectively; *E*_U_ is the unloading modulus; *σ*_1p_, *σ*_2p_ and *σ*_3p_ are the three principal stresses at the point of peak strength, while *σ*_1B_, *σ*_2B_ and *σ*_3B_ are the three principal stresses relevant to point B.

Hence, the following relation exists:

2.10 $$W=U^{\mathrm{pre}}+U^{\mathrm{post}}=U^{e}+U^{f}$$

2.11 $$\text{and}\;\;\;\;f^{f}=\frac{U^{f}}{U}=\frac{U^{\mathrm{pre}}+U^{\mathrm{post}}-U^{e}}{U^{\mathrm{pre}}+U^{\mathrm{post}}}.$$

#### 2.2.3. Residual strength region (beyond *ε*_r_)

Rocks in this region can still carry a certain amount of stress, which is termed as the residual strength. The work done is also transformed into both stored elastic energy and failure energy. Suppose point C is in this region (figure 2); following the same procedures detailed in the previous section enables us to obtain the following equations:

2.12 $$\begin{array}{rl} U^{\mathrm{pre}} & =\frac{1}{2E_{\mathrm{pre}}}[\sigma_{1p}^{2}+\sigma_{2p}^{2}+\sigma_{3p}^{2}-2\nu (\sigma_{1p}\sigma_{2p}+\sigma_{1p}\sigma_{3p}+\sigma_{2p}\sigma_{3p})\text{] , } \end{array}$$

2.13 $$\begin{array}{rl} U^{\mathrm{ss}} & =\frac{1}{2E_{\mathrm{post}}}\left[\begin{array}{c} \sigma_{1p}^{2}+\sigma_{2p}^{2}+\sigma_{3p}^{2}-2\nu (\sigma_{1p}\sigma_{2p}+\sigma_{1p}\sigma_{3p}+\sigma_{2p}\sigma_{3p})\\ -(\sigma_{1r}^{2}+\sigma_{2r}^{2}+\sigma_{3r}^{2})+2\nu (\sigma_{1r}\sigma_{2r}+\sigma_{1r}\sigma_{3r}+\sigma_{2r}\sigma_{3r}) \end{array}\right], \end{array}$$

2.14 $$\begin{array}{rl} U^{r} & =\frac{1}{E_{\mathrm{eq}-\text{r}}}[\sigma_{1r}^{2}+\sigma_{2r}^{2}+\sigma_{3r}^{2}-2\nu (\sigma_{1r}\sigma_{2r}+\sigma_{1r}\sigma_{3r}+\sigma_{2r}\sigma_{3r})] \end{array}$$

2.15 $$\begin{array}{rl} \text{and}\;\;\;\;U^{e} & =\frac{1}{2E_{u-\text{r}}}[\sigma_{1c}^{2}+\sigma_{2c}^{2}+\sigma_{3c}^{2}-2\nu (\sigma_{1c}\sigma_{2c}+\sigma_{1c}\sigma_{3c}+\sigma_{2c}\sigma_{3c})], \end{array}$$

where *U*^pre^, *U*^ss^, and *U*^r^ are the strain energies in the pre-peak, the strain-softening and the residual strength regions, respectively; *U*^e^ is the stored elastic energy; *E*_pre_ and *E*_post_ are the moduli in the elastic and post-peak region, respectively; and *E*_eq−r_ is the equivalent modulus in the residual strength region, which is mathematically calculated as the slope of the line crossing points of *ε*_r_ and C (figure 2). *E*_u−r_ is the unloading modulus in the residual strength region; *σ*_1r_, *σ*_2r_ and *σ*_3r_ are the three principal stresses at the point of *ε*_r_, while *σ*_1c_, *σ*_2c_ and *σ*_3c_ are the three principal stresses relevant to point C.

Similarly, the following equations can be used to calculate the fraction of the failure strain energy in the total work done:

2.16 $$W=U^{\mathrm{pre}}+U^{\mathrm{ss}}+U^{r}=U^{e}+U^{f}$$

2.17 $$\text{and}\;\;\;\;\;f^{f}=\frac{U^{f}}{U}=\frac{U^{\mathrm{pre}}+U^{\mathrm{ss}}+U^{r}-U^{e}}{U^{\mathrm{pre}}+U^{\mathrm{ss}}+U^{r}}.$$

Using equations (2.6)–(2.17), one can completely monitor the fraction of the failure energy, *f*^f^, in rocks at any state of stress. Apparently, the change of *f*^f^ is closely related to the post-failure behaviour of rocks, thus this approach could shed light on studying the floor heave mechanism in longwall gateroads. Additionally, it should be mentioned that this strain energy calculation model is not limited to rocks showing a strain-softening behaviour. In fact, the elastic–perfectly plastic and elastic, strain-hardening behaviour of rocks can also be captured by assessing the arithmetic sign and the value of *E*_post_*.* Moreover, the proposed model can also be applied for the rocks under uni- and bi-axial stress states where the corresponding principal stress terms need to be adaptive. Therefore, the principles of the proposed model are applicable for evaluating the complex deformation behaviours of rocks under various states of stress.

### 2.3. Model validation

We hypothesize that the fraction of the failure energy in the total work done, *f*^f^*,* correlates with the rock deformation. We use the laboratory measurements of two typical coal measure rocks (shale and sandstone) to test this hypothesis. Shale and sandstone samples were obtained from the floor layers in a coal mine in China. According to the test guidelines suggested by ISRM [39] and ASTM [40], the specimens were prepared with a diameter of 50 mm and length of 100 mm. A servo-controlled testing system, MTS815.02, was used to obtain the complete stress–strain curves for the specimens under four levels of confining pressures (0, 5 MPa, 15 MPa and 25 MPa). To capture the stress–strain curves in the post-peak region, the axial strain control model with a loading rate of 0.002% per second was used [41,42]. The test results are summarized in table 1.

**Table 1.** The complete stress–strain curves for the shales and sandstones under different confining pressures. These data were used to calculate the fraction of the failure energy (*f*^f^) based on equations (2.6)–(2.17) as the samples were deformed.

rock	confining pressure (MPa)	Young's modulus (MPa)	Poisson's ratio	peak strength MPa	strain at peak strength point (mm mm^−1^)	post-peak modulus^a^ (MPa)	residual strength (MPa)	strain at the onset of residual strength (mm mm^−1^)
shale	0	4022	0.207	25.4	0.00631	21 764	1.135	0.007425
	5	10 400	0.211	55.9	0.00538	10 818	18.48	0.008844
	15	10 500	0.341	80.2	0.00767	29 492	48.23	0.008759
	25	14 500	0.308	101.9	0.00702	29 391	61.26	0.008402
sandstone	0	6340	0.270	48.6	0.00766	20 400	9.706	0.009572
	5	24 400	0.289	87.4	0.00358	24 400	40.44	0.00528
	15	24 600	0.306	129.1	0.00525	14 900	85.2	0.00818
	25	17 200	0.265	170.3	0.00988	3894	160.5	0.01239

^a^The post-peak modulus was defined as the secant modulus between the peak-strength point and the onset point of the residual strength.

**Figure 3.**
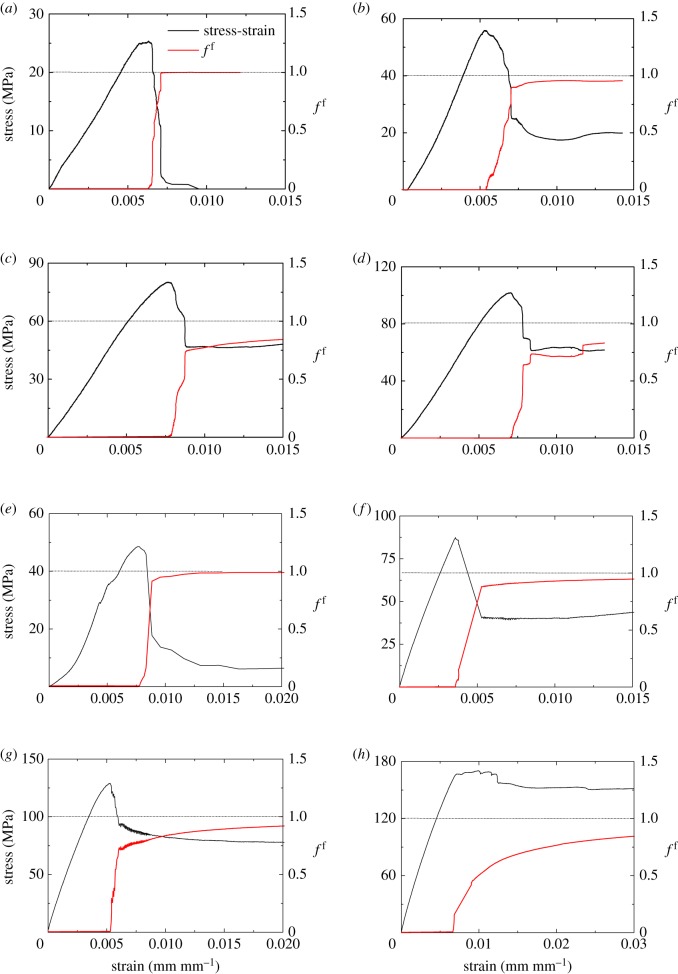
*f*^f^ versus rock deformation for shale (*a–d*) and sandstone (*e–h*) specimens under various confining pressures. (*a*) Confining pressure = 0 (shale). (*b*) Confining pressure = 5 MPa (shale). (*c*) Confining pressure = 15 MPa (shale). (*d*) Confining pressure = 25 MPa (shale). (*e*) Confining pressure = 0 (sandstone). (*f*) Confining pressure = 5 MPa (sandstone). (*g*) Confining pressure = 15 MPa (sandstone). (*h*) Confining pressure = 25 MPa (sandstone).

Substituting the data in table 1 into equations (2.6)–(2.17), the relation between *f*^f^ and the stress–strain curve of each specimen can be obtained, as shown in figure 3. For simplicity, we assumed *E*_pre_  =  *E*_u_  =  *E*_u−r_ in the calculation of *f*^f^. Figure 3 indicates that *f*^f^ is equal to zero in the pre-peak region and is drastically increased after the peak strength. It is also found that the final magnitudes of *f*^f^ show dependence on the confining pressures for both rock types. This is because the residual strength is higher for a given sample at a higher confining pressure, which tends to store more elastic energy in the sample. However, it is more important to notice that, as the sample deforms, the value of *f*^f^ monotonically increases towards a unit, which is independent of the rock types and the confining pressures. Thus, our hypothesis has been validated and *f*^f^ can serve as a reliable indicator to represent the post-failure deformation of rocks.

## 3. Floor heave mechanism: numerical simulation analysis

In this section, we use numerical simulation that is based on a finite difference method, FLAC^3D^, to discuss the floor heave mechanism of longwall gateroads. The proposed strain energy model is implemented in the numerical simulation to enable the analysis. The FLAC^3D^ model is developed based on two real longwall panels whose geological and mining conditions are presented subsequently.

### 3.1. Geological and mining conditions

**Figure 4.**
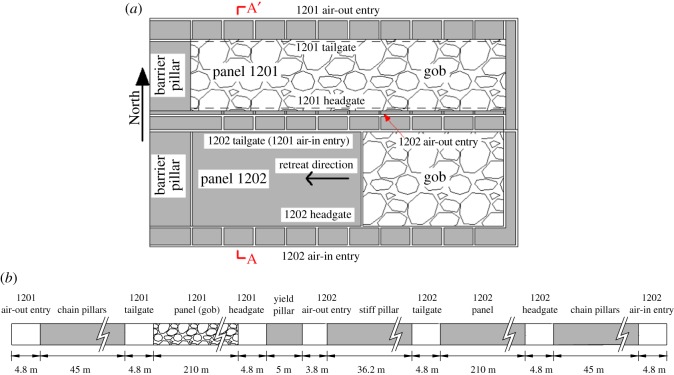
Longwall panel layout for the case study. (*a*) 1201 and 1202 panel layout. (*b*) A–A′ cross-section (location of A–A′ is shown in (*a*)).

Two longwall panels in a coal mine in China are selected for the case study. Both longwall panels extract the No. 3 coal seam which is 5.4 m thick with an average overburden depth of 455 m. The longwall with top-coal caving method was used to extract the coal seam. Roof and floor layers of the coal seam consisted of sandstone and shale, as illustrated in table 2. The shale in the immediate floor is rich in clay (mainly illite) that does not show strong swelling behaviour upon contact with water. Both panels were developed by the two-entry gateroad system, as shown in figure 4. After the completion of the retreat of panel 1201, the southernmost entry (air-in entry) of panel 1201 was reused as the tailgate of panel 1202. To meet the ventilation requirements, an air-out entry was developed in the pillars between the adjacent panels before the retreat of panel 1202. It, thereby, created a yield–stiff pillar configuration in the gateroads. The widths of the yield and stiff pillars were 5 m and 36.2 m, respectively. The dimensions of the tailgate and air-out entry of panel 1202 were 4.8 m wide by 3.6 m high and 3.8 m wide by 3.2 m high, respectively. Studies indicated that, with a proper roof and rib bolting design, this panel layout could be practically feasible without roof and pillar control problems [2].

**Table 2.** Rock strata properties used in the numerical model.

rock strata	thickness (m)	UCS (MPa)	Young's modulus (GPa)	Poisson's ratio	density (Kg m^−3^)	friction angle degree	cohesion (MPa)
shaley sandstone	22.5	35	4.3	0.25	2350	30	3.2
fine sandstone	7.5	45	8	0.23	2550	33	4.5
medium sandstone	3.7	40	6	0.22	2650	32	4.2
shale	3.2	30	1.8	0.28	2150	28	2.9
medium sandstone	3.8	40	6	0.22	2650	32	4.2
sandstone	10	38	5.8	0.23	2550	32	4.0
medium sandstone	4.3	40	6	0.22	2650	32	4.2
No. 3 coal seam	5.4	15	1.1	0.33	1400	table 3	table 3
shale	3.3	30	1.8	0.28	2150	28	2.9
medium sandstone	10.6	40	6	0.22	2650	32	4.2
shale	3	30	1.8	0.28	2150	28	2.9
medium sandstone	6.7	40	6	0.22	2650	32	4.2
fine sandstone	9	45	8	0.23	2550	33	4.5
medium sandstone	7	40	6	0.22	2650	32	4.2

**Table 3.** Rock mechanical properties of the strain-softening coal pillar.

strain (mm mm^−1^)	cohesion (MPa)	friction angle (degree)
0	1.20	22
0.01	0.45	18
1	0.45	18

**Figure 5.**
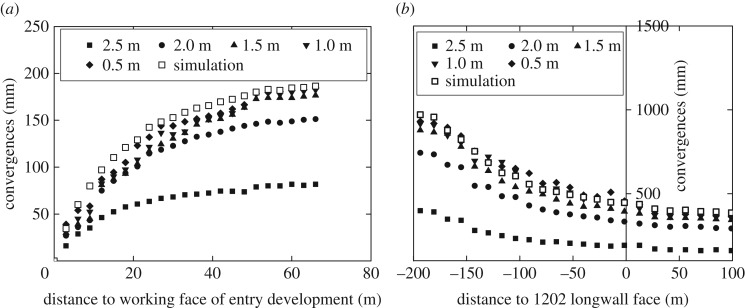
In-mine measurements of the floor heave in 1202 air-out entry. The numbers in legends represent the vertical distance below the floor surface of 1202 air-out entry. (*a*) During entry development. (*b*) During panel retreat.

Severe floor heave occurred in 1202 air-out entry during the development and retreat of panel 1202. The measurements of the floor heave are plotted in figure 5. It is found that the magnitude of floor heave is below 200 mm during entry development, while it accumulates to over 900 mm after panel retreat. Therefore, the floor heave mainly occurred during the retreat of panel 1202. In addition, it is noticed that the accumulation of floor heave is mainly contributed by the deformation of the rocks in the shallow part of the immediate floor. For instance, the deformation of the rocks within 2 m below the floor surface accounts for 80% of the total floor heave, which indicates the massive floor heave is dominated by the post-failure deformation of the floor rocks.

### 3.2. Numerical analysis

**Figure 6.**
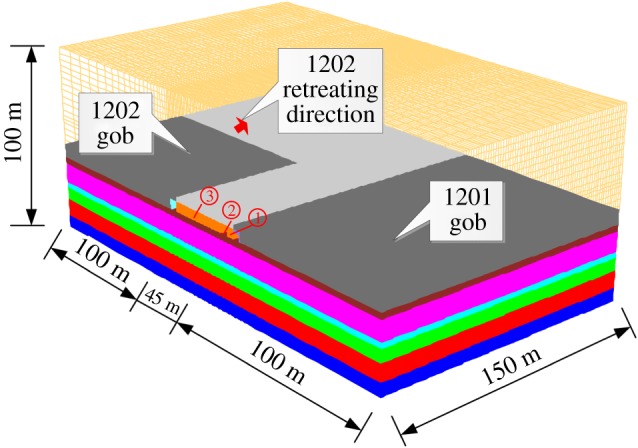
3D numerical model for the floor heave analysis. ①: Yield pillar; ②: 1202 air-out entry; ③: Stiff pillar.

To understand the mechanism of the floor heave in longwall gateroads during mining operations, a finite difference model which is based on FLAC^3D^ was developed. The 3D model consisted of one-half each of panels 1201 and 1202 and the gateroad system between them. The dimension of the model is 245 × 150 × 100 m, as shown in figure 6. The *in situ* stresses of the No. 3 coal seam are: *σ*_v_  =  11.7 MPa, *σ*_H_  =  14.1 MPa and *σ*_h_  =  6.3 MPa [43]. The horizontal and bottom sides were roller-constrained. The Mohr-Coulomb failure criterion was used to simulate the rock strata except for the pillar and gob materials.

We use the strain-softening criterion to capture the realistic mechanical behaviour of the coal pillars [44]. The other rock layers including the immediate roof/floor are assumed to follow the Mohr-Coulomb instead of the strain-softening criterion. We do this because too many uncertainties may be involved in the model calibration if more than one strain-softening material is considered. In other words, it helps us to significantly reduce the difficulties in the model calibration by assuming a Mohr-Coulomb immediate floor. Tables 2 and 3 present the calibrated rock mechanical properties for the rock layers and pillar model. The agreement between the measured and simulated floor heaves in figure 5 supports the calibrated properties in the model. In addition, the gob rocks show strain-hardening behaviour [45], whose constitutive equation has been studied previously [2]:

3.1 $$\sigma =\frac{E_{0}\varepsilon }{\left(1-\varepsilon /\varepsilon_{m}\right)}\text{, }$$

where *ε*_m_  =  (*b* − 1)/*b* and $E_{0}=10.39\sigma_{c}^{1.042}/b^{7.7}$. *ε*_m_ is the maximum strain of the gob material. *b* is the bulking factor of the gob, which depends on the height of roof caving and is equal to 1.2–1.25 [2]. *σ*_c_ and *E*_0_ are the *in situ* vertical stress and the initial modulus of the gob material, respectively. We use the double-yield model in FLAC^3D^ to capture the constitutive relation shown in equation (3.1). The double-yield model requires two groups of inputs: the initial material properties and the cap pressures which are associated with the total strains. The capture pressures basically determine the characteristics of the strain-hardening behaviour in the simulation. A trial-and-error approach is required to match the stress–strain curve of the double-yield model to that defined by equation (3.1). The determined mechanical properties of the gob material are given in tables 4 and 5.

**Table 4.** Material properties of the gob model.

density (kg m^−3^)	bulk modulus (GPa)	shear modulus (GPa)	friction angle (degree)	dilation angle (degree)
1000	8.69	6.35	20	5

**Table 5.** Cap pressures of the gob model.

strain (mm mm^−1^)	0	0.01	0.02	0.03	0.04	0.05	0.06	0.07
stress (MPa)	0	0.23	0.49	0.79	1.13	1.54	2.01	2.58
strain (mm mm^−1^)	0.08	0.09	0.10	0.11	0.12	0.13	0.14	0.15
stress (MPa)	3.28	4.15	5.27	5.96	6.76	11.99	17.22	27.67

**Figure 7.**
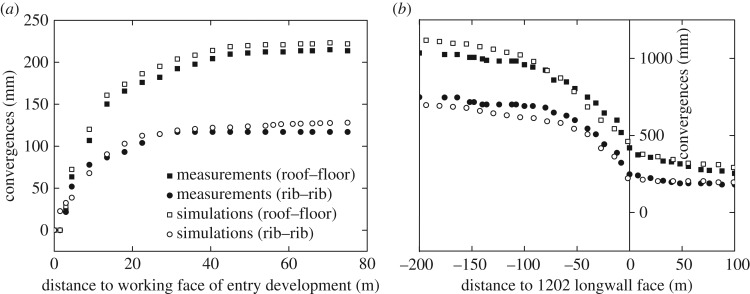
Comparison of the entry convergences between the measurements and the numerical simulations. The agreement between the measurements and simulations supports the calibrated numerical model. (*a*) During entry development. (*b*) During panel retreat.

Figure 5 shows the agreement between the simulated and the measured floor heaves. For further model validation, we also compare the measured and simulated roof–floor (and rib–rib) convergence in figure 7. The agreement between the in-mine measurements and simulation results, as illustrated in figures 5 and 7, supports the calibrated properties in the numerical model.

To evaluate the mechanism of the floor heave in 1202 air-out entry, the proposed model for calculating the fraction of the failure strain energy, *f*^f^, was implemented in the numerical simulation. In each time step of the numerical modelling, *f*^f^ of each element was calculated and updated until the mechanical equilibrium was reached. Note that the model was solved as the actual sequence of mining operations in the field, i.e. panel 1201 was first developed and retreated, followed by the development and retreat of panel 1202.

**Figure 8.**
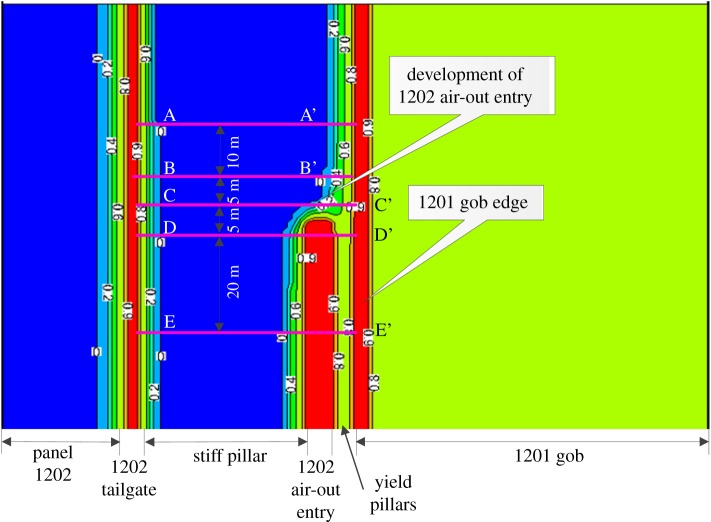
Contour of *f*^f^ in the immediate floor during the development of the 1202 air-out entry.

**Figure 9.**
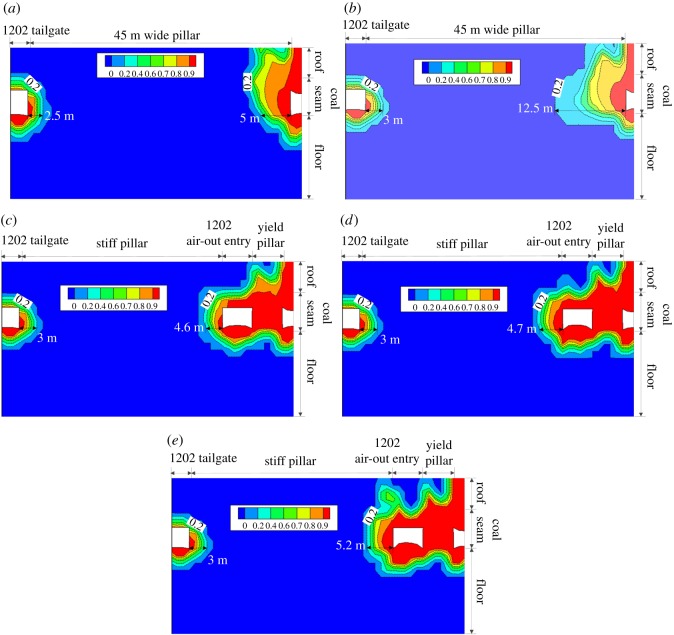
Contour of *f*^f^ in the surrounding rocks of the 1202 air-out entry during development. These cross-sections are 30 m high by 50 m wide, and the contour lines show the *f*^f^ distribution due to the influence of the mining abutment pressures (not the boundary condition effect since the model dimension is 245 × 150 × 100 m). (*a*) A–A′ cross-section (*b*) B–B′ cross-section. (*c*) C–C′ cross-section. (*d*) D–D′ cross-section. (*e*) E–E' cross-section.

Figure 8 presents the plane view of the contour of *f*^f^ in the immediate floor during the development of the 1202 air-out entry. Warm colours represent the rocks with larger *f*^f^. Owing to the loss of vertical constraint after development, the immediate floor of the 1202 air-out entry shows *f*^f^ larger than 0.9. To further evaluate the process of floor heave, five reference locations, i.e. 15 m, 5 m and 0 m ahead of the developing face as well as 5 m and 25 m behind the developing face, were marked (figure 8). A cross-section view of the *f*^f^ distribution at each reference location is given in figure 9. As the developing face approaches, the *f*^f^ in the immediate floor accumulates gradually, as shown in figure 9*b*. After development, the *f*^f^ in the yield pillar is larger than 0.9 and the regions with *f*^f^  ≥  0.9 in the immediate floor slightly propagate towards the stiff pillar on the left-hand side. Such characteristics of the *f*^f^ distribution during the entry development demonstrate that the massive deformation of the post-failure rocks in the immediate floor initiates at the yield pillar side and mainly moves to the stiff pillar side. The complex distribution of *f*^f^ in the cross-sectional plots in figure 9 is caused by the longwall abutment pressures, not by the boundary conditions, considering the model dimensions are large enough (245 × 150 × 100 m).

**Figure 10.**
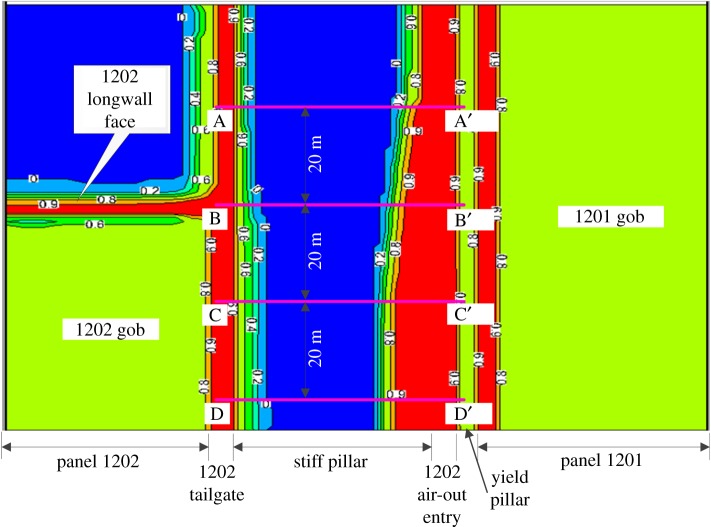
Contour of *f*^f^ in the immediate floor during the retreat of panel 1202.

**Figure 11.**
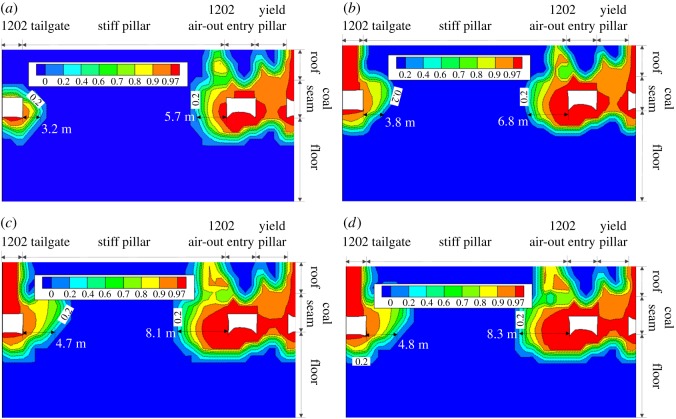
Contour of *f*^f^ in the surrounding rocks of the 1202 air-out entry during panel retreat. These cross-sections are 30 m high by 50 m wide, and the contour lines show the *f*^f^ distribution due to the influence of the mining abutment pressures (not the boundary condition effect since the model dimension is 245 × 150 × 100 m). (*a*) A–A′ cross-section. (*b*) B–B′ cross-section. (*c*) C–C′ cross-section. (*d*) D–D′ cross-section.

Similarly, the plane view of the contour of *f*^f^ in the immediate floor during the retreat of panel 1202 is shown in figure 10. Warm colours also represent the rocks with larger *f*^f^. Because the weight of the upper rocks is borne by the chain pillars after the retreat of panel 1202, domains with larger *f*^f^ expand on both sides of the chain pillars. Note that a more significant influence on the *f*^f^ distribution is observed on the right-hand side of the chain pillars, which is probably due to the deteriorated coal and rock properties after the effect of the previous mining operations. To further evaluate the process of the floor heave in the longwall retreating period, four reference locations, i.e. 20 m and 0 m ahead of as well as 20 m and 40 m behind the 1202 longwall face, were marked (figure 10). A cross-section view of the *f*^f^ distribution at each reference location is given in figure 11. As the longwall face approaches, a slight increase of *f*^f^ in the immediate floor of the 1202 air-out entry is observed near the stiff pillar, as shown in figure 11*a,b*. Behind the longwall face, however, the contour lines of *f*^f^  ≥  0.9 in the entry floor significantly propagate towards the stiff pillar, resulting in a more severe floor instability issue. Meanwhile, no significant propagation of *f*^f^ is observed in the vertical direction. The characteristics of the *f*^f^ distribution suggest that, during panel retreat, massive deformation of the post-failure floor rocks occurs near the stiff pillar side.

In summary, the massive floor heave in the longwall gateroad initiates from the rib taking less loads (yield pillar) and the rock deformation propagates towards the other rib, which bears greater loads (stiff pillar). Knowing the floor heave mechanism enables us to propose effective strategies to control the floor heave in longwall gateroads. For instance, during the entry development, installing standing supports close to the yield pillar may mitigate the floor heave initiation because the standing supports reduce the loads taken by the yield pillar. During the retreat of panel 1202, the standing supports can be installed close to the stiff pillar side of the entry rib to reduce the propagation of the floor heave towards the deeper surrounding rocks. Moreover, floor bolts may also be effective to control the floor heave as the deformation accumulation (*f*^f^) is mainly caused by the shallow parts of the floor layers (figure 11).

## 4. Conclusion

The objective of this study was to evaluate the floor heave mechanism in longwall gateroads based on strain energy analysis that accounts for the post-peak deformation of rocks. For this purpose, we developed an analytical strain energy model to calculate the fraction of the failure energy in the total work done on the entry floor by the mining-induced loads. This model enables us to assess the accumulated deformation of the post-failure rocks, which is the major contributor to the massive floor heave in the longwall gateroads.

The strain energy model was validated based on rock mechanical measurements. We then implemented the strain energy model into a finite difference package, FLAC^3D^, to study the floor heave mechanism based on a case study. The simulated longwall entry employed a stiff–yield pillar configuration. It has been observed that the massive deformation of the post-failure rocks in the immediate floor starts from the rib taking less loads (yield pillar) and eventually moves to the other rib transmitting significant amount of loads (stiffness pillar). The corresponding implications on control strategies of floor heave were discussed.

In addition, the proposed strain energy model can also be applied to other ground control problems where failure is mainly caused by the post-peak behaviour of rocks, such as the squeezing failure in longwall gateroads [46] and bleeder pillar instability [47]. Such issues may not be satisfactorily addressed if only the peak strength of rock is considered.

## Data accessibility

The datasets from this study are publicly available on the Dryad Digital Repository (http://dx.doi.org/10.5061/dryad.s4302j0) [48].
